# Evolution of Avian orthoavulavirus 16 in wild avifauna of Central Asia

**DOI:** 10.1016/j.heliyon.2019.e03099

**Published:** 2020-01-07

**Authors:** Kobey Karamendin, Aidyn Kydyrmanov, Yermukhammet Kasymbekov, Aigerim Seidalina, Klara Daulbayeva, Marat Sayatov, Sasan Fereidouni

**Affiliations:** a Laboratory of Viral Ecology, Institute of Microbiology and Virology, 103 Bogenbay Batyr Str, 050010, Almaty, Kazakhstan; b Research Institute of Wildlife Ecology, University of Veterinary Medicine Vienna, Austria

**Keywords:** Microbiology, Virology, Avian paramyxovirus, Avian orthoavulavirus, AOAV-16, Next-generation sequencing, Evolution, Kazakhstan

## Abstract

In 2014, a novel Avian orthoavulavirus 16 species was described among wild birds in Korea. In 2018, after massive parallel sequencing of archival strains of Avian orthoavulaviruses, isolated in 2006 in Central Kazakhstan, isolates belonging to this serotype were detected. The obtained data allowed to trace the evolution of this serotype in Asia and to reveal its evolutionary relationships with other *Avulavirinae* subfamily species. It was determined that Avian orthoavulavirus 16 is phylogenetically very close to Avian orthoavulavirus 1 (Newcastle disease virus) in its genomic characteristics. It is known that Avian orthoavulavirus 1 is divided into two phylogenetically distant Classes I and II. Avian orthoavulavirus 16 turned out to be very close to lentogenic Class I, which circulates mainly among wild birds. It was suggested that Avian orthoavulaviruses 1 and 16 may have common evolutionary origin and in ecological terms, both serotypes are circulating among wild birds of the order Anseriformes (ducks and geese), but Avian orthoavulavirus 1 has gradually replaced Avian orthoavulavirus 16 from active circulation.

## 1 Introduction

Avian paramyxoviruses or, according to the new classification, Avian meta-, para- and orthoavulaviruses (AOAV) belong to the subfamily *Avulavirinae of Paramyxoviridae* family, possessing linear negative-sense single-stranded RNA. The subfamily *Avulavirinae* currently contains twenty species (Avian meta-, para- and orthoavulaviruses 1–20) (International Committee on Taxonomy of Viruses, ICTV) based on hemagglutination inhibition (HI) assay and genetic analyses.

The *Paramyxoviridae* family genome consists of six genes encoding the following proteins: nucleocapsid protein (NP); phosphoprotein (P); matrix protein (M); fusion protein (F); hemagglutinin-neuraminidase (HN) and an RNA-dependent RNA polymerase (L), as well as two non-structural proteins V and W (Lamb Robert and Parks Griffith, 2013). Avian metaavulavirus 6 possesses an additional small hydrophobic (SH) gene that is absent in other *Avulavirinae* subfamily representatives (Wilson et al., 2006). AOAV-1 (Newcastle Disease Virus) is one of the most threatening pathogens for poultry and causes significant economic loss. Other avulaviruses are less pathogenic but can potentially cause infection of respiratory or intestinal tracts of birds with varying degree of pathogenicity (Kim et al., 2012).

The recently discovered novel AOAV-16 strain AOAV-16/WB/Korea/UPO216/2014 was isolated from a wild bird in Korea in 2014 and was further approved as a reference strain for this genotype (ICTV, Lee et al., 2017). The archival strain under study AOAV-16/white fronted goose/Central Kazakhstan/1791/2006 was isolated from a wild goose in Kazakhstan in 2006. Upon its isolation in 2006, this strain was erroneously identified as AOAV-1 in a HI assay. When the complete genome sequence of the virus was obtained, its homology with the newly discovered AOAV-16 genotype was revealed.

In this paper we present the genetic analysis of an AOAV-16 isolate that was identified eight years before the Korean isolate; therefore its evolutionary history may increase our knowledge about ecology of this genotype. As AOAV-1 and 16 are the antigenically and genetically most closely related (Karamendin et al., 2017; Aziz-Ul-Rahman et al., 2018) among all avulaviruses, we conducted a comparative genetic analyses that may elucidate their evolutionary relationships.

## 2 Materials and methods

### 2.1 Sample collection

Cloacal and tracheal swabs and fresh feces were collected in Central Kazakhstan in 2006. The samples were collected using sterile swabs (F.L. Medical, Italy) and stored in vials with viral transport medium containing Dulbecco's Modified Eagle's Medium (Sigma-Aldrich, USA), antibiotics (2000 U/ml penicillin, 2 mg/ml streptomycin, 50 μg/ml gentamycin), antimycotic (50 U/ml nystatin) and 0.5% bovine serum albumin. All procedures involving sampling of wild birds were conducted in concordance with Rules for Conducting Biomedical Experiments, Preclinical (Non-Clinical) and Clinical Studies (No. 697, 12 November 2007, Republic of Kazakhstan), and were approved by the Institute of Microbiology and Virology Local Ethics Committee (Approval Number: #02-09-60 from 1 October 2019).

### 2.2 Virus isolation

Viral RNA was extracted from the samples using QIAamp Viral RNA Mini kit (Qiagen, Hilden, Germany) according to the manufacturer's instructions. The RNA was screened by RT-PCR targeting the M-gene of the avian influenza viruses (AIV), and AIV-negative samples were inoculated into 10-day-old embryonated chicken eggs (ECE) and then incubated for 72 h at +36°С (WHO, 2002). The allantoic fluid was checked for presence of the hemagglutinating viruses using hemagglutination assay with 0.75 % chicken erythrocytes.

### 2.3 Production of rabbit antiserum

An antiserum to the AOAV-16/white-fronted goose/Central Kazakhstan/1791/2006 strain was raised by double immunization of rabbits with the purified ultra-centrifuged viral suspension. The first immunization was conducted by intracutaneous injections of the viral suspension mixed with complete Freund's. A second immunization was conducted intravenously with incomplete adjuvant after three weeks. Antiserum was collected 7–14 days after the second immunization (Saiatov et al., 1985).

### 2.4 Hemagglutination inhibition (HI) assay

A standard HI assay (Manual of Diagnostic Tests and Vaccines for Terrestrial Animals, OIE, 2010) was conducted using antisera specific to the AOAV 1–9 reference strains.

### 2.5 Sequencing and data analysis

RT-PCR assays were performed on the basis of one step protocols using AccessQuick One-Step RT-PCR Kit (Promega, USA) employing pan-PMV primers targeting the conserved fragment of L-gene (Tong et al., 2008). The PCR products were Sanger-sequenced using the same PCR primers on an ABI 3730xl DNA analyzer (Applied Biosystems, USA) using BigDye Terminator Kit v.3.1 Sequencing Kit (Applied Biosystems, USA).

For complete genome sequencing, viral RNA was used as a template for library preparation using the NEBNext Ultra RNA Library Preparation and rRNA Depletion kits (New England Biolabs, USA) according to manufacturer's protocol. Library size selection was performed using Ampure XP beads (Beckman Coulter, USA). The size of the library was determined on a Bioanalyzer 2100 (Agilent Technologies, USA) using a High Sensitivity DNA Chip. Paired-end sequencing was performed on an Illumina MiSeq instrument using a MiSeq reagent kit v3 (Illumina, USA). Sequence data were assembled and analyzed using Geneious 11.0 software (Biomatters, New Zealand).

### 2.6 Phylogenetic and divergence analyses

Nucleotide and amino-acid sequences were aligned and their evolutionary distances estimated using Mega 6.0 software (Tamura et al., 2013). Twenty-three other full-length AOAV genome sequences representing other AOAV genotypes from Genbank database were used as references for this analysis. A maximum-likelihood phylogenetic tree for complete genome sequences was constructed in MEGA 6.0 using (2 categories + G, parameter = 1.6599) with 500 bootstrap replicates. Phylogenetic tree based on the aligned AOAV-1 and 16 NP genes was inferred during molecular clock analyses in BEAST 2.6.0 (Bouckaert et al., 2019) using a GTR model with gamma and invariant sites.

Estimates of evolutionary divergence were conducted using the JTT matrix-based model (Jones et al., 1992). The rate variation among sites was modeled with a gamma distribution (shape parameter = 2). The analysis involved 21 amino acid sequences. All positions containing gaps and missing data were eliminated. There were a total of 328 positions in the final dataset. Evolutionary analyses were conducted in MEGA 6.

### 2.7 Recombination analysis

Potential recombination events in complete genomes of AOAV-16 from this study and Korean strain were sought using seven algorithms (RDP, GENECONV, Chimaera, MaxChi, BootScan, SiScan and 3Seq) within the RDP4 package (Martin et al., 2015).

### 2.8 Selection pressure analysis

Site-specific selection pressure for individual AOAV-16 genes were measured as the ratio of nonsynonymous (dN) to synonymous substitutions (dS) using the four codon-based maximum likelihood approaches: single likelihood ancestor counting (SLAC), fixed effects likelihood (FEL), mixed effects model of evolution (MEME), and fast unbiased Bayesian approximation (FUBAR) available at the Datamonkey facility (Pond and Frost, 2005). BUSTED (Branch-Site Unrestricted Statistical Test for Episodic Diversification) approach also available at the datamonkey web server (https://www.

datamonkey.org/) was used to asses if a gene has experienced a positive selection at any site at the gene-wide level.

### 2.9 Molecular clock estimations

Bayesian time scaled analyses were conducted by the Bayesian Markov Chain Monte Carlo (BMCMC) method implemented in BEAST v2.6.0 program utilizing a nucleoprotein gene sequence dataset.

The tests were run for 50 × 10^6^ generations sampling every 1000 steps. The data was analyzed using the GTR + C6+I substitution model, using relaxed lognormal molecular clock. Bayesian coalescent exponential population size tree prior was used to describe the demographic history. The convergence of BEAST output file was estimated in TRACER v1.7.1 where statistical uncertainty was provided by the 95% HPD values. The maximum clade credibility tree was generated using Tree Annotator v 2.5.1 (from BEAST package) and visualized in FigTree v1.4.4.

## 3 Results

### 3.1 Sample collection and preliminary virus identification

In the first description of the reference strain from Korea (Lee et al., 2017), it was not exactly known about the main host range of AOAV-16. Below is the species composition of the birds studied in 2006, from which both AOAV-1 and AOAV-16 serotypes were simultaneously isolated. The samples were collected from three important regions in Kazakhstan where migrating birds concentrate: Korgaljyn lakes system in central Kazakhstan, West coast of the Caspian Sea and wetlands of southern Kazakhstan. In total, 652 samples from 509 wild aquatic and terrestrial birds representing 44 avian species of nine orders were collected. The detailed list of samples is represented in Table 1.

**Table 1:** Samples collected from wild birds in Kazakhstan in 2006.

Order/Species (Latin)	Species (English)	Number of		AOAV Isolate
		birds	samples	
*Anseriformes*				
Anser anser	Greylag Goose	30	33	AOAV-16/greylag goose/Central Kazakhstan/1790/2006
Anser albifrons	White-fronted Goose	8	8	AOAV-16/white fronted goose/Central Kazakhstan/1791/2006 and AOAV-16/white fronted goose/Central Kazakhstan/1792/2006
Cygnus olor	Mute swan	9	12	
Cygnus cygnus	Whooper Swan	1	1	
Tadorna ferruginea	Ruddy Shelduck	5	5	AOAV-16/ruddy shelduck/Central Kazakhstan/1787/2006
Tadorna tadorna	Common Shelduck	2	2	
Anas platyrhynchos	Mallard	34	43	
Anas crecca	Teal	43	54	
Anas strepera	Gadwall	26	37	
Anas penelope	Eurasian Wigeon	24	29	AOAV-1/wigeon/Central Kazakhstan (Korgalzhyn)/1807/2006, Class I and AOAV-1/wigeon/Central Kazakhstan (Korgalzhyn)/1819/2006, Class I
Anas acuta	Northern Pintail	15	16	AOAV-1/pintail/Central Kazakhstan (Korgalzhyn)/1786/2006, Class I
Anas querquedula	Garganey	3	5	
Anas clypeata	Shoveler	6	10	
Netta rufina	Red-crested Pochard	8	15	
Aythya ferina	Common Pochard	21	30	AOAV-1/common pochard/Central Kazakhstan (Korgalzhyn)/1812/2006, Class I
Aythya nyroca	Ferruginous Duck	1	1	
Aythya fuligula	Tufted Duck	3	5	
Bucephala clangula	Common Goldeneye	4	4	
Mergus albellus	Smew	1	1	
Mergus merganser	Goosander	1	2	
*Charadriiformes*				
Himantopus himantopus	Black-winged Stilt	2	4	
Calidris minuta	Little Stint	1	2	
Limosa lapponica	Bar-tailed Godwit	1	1	
Larus ichthyaetus	Great Black-headed Gull	12	15	
Larus ridibundus	Black-headed Gull	30	34	
Larus genei	Slender-billed Gull	7	9	
Larus argentatus	Herring Gull	42	58	
Larus cachinnans	Yellow-legged Gull	7	11	
Larus canus	Common Gull	8	9	
Chlidonias leucopterus	White-winged Tern	1	2	
Hydroprogne caspia	Caspian Tern	7	14	
Thalasseus sandvicensis	Sandwich Tern	1	1	
Sterna hirundo	Common Tern	39	46	
*Gruiformes*				
Gallinula chloropus	Moorhen	1	1	
Fulica atra	Common Coot	68	81	
*Columbiformes*				
Columba livia	Rock Dove	3	3	
*Galliformes*				
Phasianus colchicus	Pheasant	2	2	
*Phoenicopteriformes*				
Phoenicopterus roseus	Greater Flamingo	1	2	
*Podicipediformes*				
Podiceps grisegena	Red-necked Grebe	1	2	
Podiceps cristatus	Great Crested Grebe	2	4	
*Suliformes*				
Phalacrocorax carbo	Cormorant	20	26	
Pelecanus onocrotalus	Great White Pelican	4	8	
*Ciconiiformes*				
Ardea alba	Great White Egret	3	3	
Ardea cinerea	Grey Heron	1	1	
Total		509	652	

Preliminary identification of hemagglutinating agents was conducted in the HI assay, which revealed that all of them belonged to the AOAV-1 genotype (data not shown). So, positive results were obtained exclusively from wild ducks and geese from the same region in central Kazakhstan.

### 3.2 Determination of the complete genome sequence of AOAV-16 strain and comparative molecular analyses

The complete sequence of AOAV-16/white-fronted goose/Central Kazakhstan/1791/2006 is available at Genbank under the accession number MH423285. The obtained nucleotide sequences were translated into amino acid (aa) sequences and BLAST similarity search was conducted (Table 2).

**Table 2:** Amino acid identity between AOAV-1 and 16 by six genes.

Gene of AOAV-16	AOAV-1 BLASTp Results					
	% identity	Most Similar AOAV-1 Strain	Class	Genotype	Pathotype	Accesion Number
NP	80	AOAV-1/CK/GX/65/2015	I	Ib	Lentogenic	MH274992
P	57	AOAV-1/chicken/U.S./101250–2/2001	I	Ic	Lentogenic	AY626268
M	79	AOAV-1/Mallard/Oregon/13685/2009	I	Ic	Lentogenic	JN942056
F	79	AOAV-1/northern pintail/AK/44493–779/2009	I	Ic	Lentogenic	KC503464
HN	70	AOAV-1/Sheldrake duck/China/Guizhou/02/2016	I	Ib	Lentogenic	KX602323
L	77	AOAV-1/Yakutiya/mallard/852/2011	II	Ib	Lentogenic	KJ920203

A comparative study for six genes revealed the greatest similarity between the NP, M, F and L genes, with 77–80% identity. For the HN-gene, they were identical to a lesser extent (70%), and the least similarity was registered for the P-gene (57%). The greatest genetic variability in the P gene had been previously observed in earlier studies of avulaviruses 1, 8, and 20 (Kattenbelt et al., 2006; Fereidouni et al., 2018; Karamendin et al., 2019). Obtained results suggest that all proteins of AOAV-16 were more similar to lentogenic variants of AOAV-1 than to velogenic. It is interesting that the highest similarity was found to the almost simultaneously circulated AOAV-1 strains, not to ‘old’ strains.

Estimates of the evolutionary divergence of the amino acid sequences of the most variable P protein of all APMVs showed highest relatedness of the APMV-16 Kazakh and Korean strains to APMV-1 with values of 0.558 and 0.553, respectively. This index is the lowest among all *Aulavirinae* species, compared to 2.987 (highest) between APMV 4 and 5 (Supplementary Table S1). These data are consistent with previous study where closest relatedness between AOAV-16 and AOAV-1 was observed by the whole genome (Lee et al., 2017) and by F and HN genes (Karamendin et al., 2017).

To understand deeper the molecular basis of the similarity of these serotypes, we conducted a comparative study of their genomes (Table 3). Nucleotide sequences of strains AOAV-16/white fronted goose/Central Kazakhstan/1791/2006, AOAV-16/WB/Korea/UPO216/2014) and lentogenic AOAV-1 were taken for analysis.

**Table 3:** Comparative genome characterization of the isolate AOAV-16/white fronted goose/Central Kazakhstan/1791/2006 with Korean AOAV-16 and AOAV-1 lentogenic Class I strains.

Gene	Strain	Gene length (nt)	ORF length (nt)	Protein length (aa)	Gene Start	Gene End
NP	KZ strain	1,758	1,476	491	ACGGGTAGAA	TTAGAAAAAA
	KR strain	1,758	1,476	491	ACGGGTAGAA	TTAGAAAAAA
	APMV-1	1,746	1,470	489	ACGGGTAGAA	TTAGAAAAAA
P	KZ strain	1,475	1.200	399	ACGGGTAGAA	TTAAGAAAAAA
	KR strain	1,475	1,200	399	ACGGGTAGAA	TTAAGAAAAAA
	APMV-1	1,463	1,200	399	ACGGGTAGAA	TTAAGAAAAAA
V	KZ strain	–	–	245	–	–
	KR strain	–	–	245	–	–
	APMV-1	–	–	245	–	–
W	KZ strain	–	–	–	–	–
	KR strain	–	–	140	–	–
	APMV-1	–	–	183	–	–
M	KZ strain	1,242	1,095	364	ACGGGTAGAA	TTACAAAAAAA
	KR strain	1,242	1,095	364	ACGGGTAGAA	TTACAAAAAAA
	APMV-1	1,241	1,095	364	ACGGGTAGAA	TTAGAAAAAA
F	KZ strain	1,823	1,656	551	ACGGGGAGAA	TTAGAAAAAAA
	KR strain	1,823	1,656	551	ACGGGGAGAA	TTAGAAAAAAA
	APMV-1	1,792	1,662	553	ACGGGTAGAA	TTAGAAAAAA
HN	KZ strain	2,041	1,857	618	ACGGGGAGAA	TTAGAAAAAA
	KR strain	2,023	1,857	618	ACGGGGAGAA	TTAGAAAAAA
	APMV-1	2,001	1,851	616	ACGGGTAGAA	TTAGAAAAAA
L	KZ strain	6,734	6,609	2,202	ACGGGGAGAA	TTAAGAAAAAA
	KR strain	6,734	6,609	2,202	ACGGGGAGAA	TTAAGAAAAAA
	APMV-1	6,703	6,615	2,204	ACGGGTAGGA	TTAGAAAAAA

AOAV-16/white-fronted goose/Central Kazakhstan/1791/2006 (KZ strain) and AOAV-16/WB/Korea/UPO216/2014) is Korean strain (KR strain). Varying positions are underlined.

Genome sizes of AOAV-1 and 16 were the closest among all avulaviruses 1–20. The complete genome sequence of the Kazakh AOAV-16 strain constituted 15198 nt, which corresponds to the length of AOAV-1 of Class I; while the strain AOAV-16/WB/Korea/UPO216/2014 (Genbank Accession Number KY511044) had 15180 nt, which is six nucleotides shorter than the genetically closest AOAV-1/La Sota strain (15186 nt) belonging to the genotype II of Class II.

The lengths of the leader and trailer sequences in AOAV-16 were 55 nt and 47 nt respectively, which is conserved in this genotype (Samal, 2011), and AOAV-1 had lengths of 55 nt and 114 nt. There were slight variations in the lengths of the individual coding regions and gene-end (GE) sequences of AOAV-1 and 16, but overall a high degree of similarity was observed. The proposed gene-start (GS) and GE intergenic signal sequences (IGS) are relatively conserved among AOAV-1 and 16 genotypes and had few variations. Nucleotide quantity in IGS also varied: 2,1,1,31,48 in AOAV-1; 2,1,6,0,14 in Korean AOAV-16 strain and 2,1,6,0,32 in Kazakh AOAV-16 strain.

Both Kazakh and Korean strains may have originated from a common ancestor, and therefore the lengths of the ORFs were identical. However, during evolutionary processes, the Korean strain has lost 18 nucleotides in 5′ non-coding region of the HN gene. ORF of different genes of AOAV-1 strains differed from AOAV-16 by 6–12 nt in NP,P,F and L genes. The biggest difference in size was observed by the HN gene of AOAV-1 that contained 141 nt less than AOAV-16. A high degree of HN gene size variation is characteristic of AOAV-1. It is known that AOAV-1 HN protein of 616 aa has been found in the lentogenic but not in velogenic NDV strains, whereas the HN protein of 571 aa can only be detected in velogenic strains (Römer-Oberdörfer et al., 2003). The length of the HN protein of the Kazakh AOAV-16 was 618 aa, which is similar to that of lentogenic viruses.

In order to assess the possibility of genetic recombination between AOAV-16 and AOAV-1 we have conducted the respective analysis using the RDP4 program package. Obtained results has revealed no obvious recombination events between these two serotypes. This is not surprising as recombinations are generally rare or even absent in most negative sense RNA viruses (Han and Worobey, 2011).

During the genetic characterization of all avulaviruses 1–20, it is common to study conserved regions in different parts of the genome (Aziz-Ul-Rahman et al., 2018).

### 3.3 Internal proteins analysis

The study of conserved sites for internal genes (Supplementary Figure S1) showed that all sites in the NP, P, L genes showed high degree of similarity.

The nucleoprotein (NP) gene of KZ and KR AOAV-16 strains was 1758 nt long and encoded 491 amino acids (aa). The Kazakh and Korean strains differed in two amino acids V100A and I111V.

The motif F-X4-YX3-Φ-S-Φ-A-M-G, where X is any aa and Φ is any aromatic aa, has been identified in all members of the family *Paramyxoviridae* and considered responsible for N–N-self-assembly during genomic RNA binding (Morgan, 1991). By this motif, both AOAV-1 and 16 strains had NP containing identical sequence segment 322-FAPAEYACLYSFAMG-336 and had Y333F substitution.

The sites of extensive divergence were revealed in the phosphoprotein (P) gene of AOAV-16, where 21 non-synonymous substitutions were found (Table 4). The P gene was 1475 nt long and encoded a protein with 399 aa. The P protein of the KZ strain shares an aa sequence identity of 94.7 % with the Korean strain and is considered the most variable protein. The P-gene of KZ and KR AOAV-16 strains contained a putative-editing site AAAAAAGGG at positions 394–402. The addition of a single G-residue to the encoded mRNA would produce a V mRNA encoding a 245-aa V-protein in both strains, but the addition of two G-residues would produce a W mRNA encoding a 140 aa W-protein only in the Korean strain, while the W mRNA of the KZ strain is apparently not encoded, as it gives only one aa after editing site. The P gene of AOAV-1 contained an identical putative editing site AAAAAGGG at the same 394–401 positions. The addition of a single G residue to the encoded mRNA would produce a V mRNA encoding a 245 aa V protein. The addition of two G residues would produce a W mRNA encoding a 183 aa W protein (Steward et al., 1993). This V-protein domain of both genotypes contains the conserved cysteine rich motif that is characteristic of most members of the genus *Orthoavulavirus* (Sun et al., 2004).

**Table 4:** Amino acid substitutions in proteins of AOAV-16 genotype.

	Viral protein																													
	NP		P																					M	F					
Position	100	111	55	62	65	67	72	95	98	135	140	147	149	151	152	159	171	173	196	217	237	321	377	355	18	24	67	458	4 8 7	504
KZ strain	V	I	T	R	G	E	V	P	E	L	P	T	H	A	T	A	I	P	P	H	A	T	A	N	A	R	I	N	D	V
KR strain	I	V	A	K	D	D	A	L	G	S	L	M	Y	V	A	V	M	L	L	Y	T	A	V	T	T	Q	V	S	E	I
	L																						HN							
Position	71	220	276	389	400	456	492	498	787	890	1332	1335	1408	1443	1610	1886	1908	2057	2071	2137	2173	2183	69	113	146	201	225	301	407	440
KZ strain	R	D	V	I	V	V	A	K	T	D	M	V	S	I	N	V	V	A	M	I	I	A	L	Q	I	R	H	G	V	A
KR strain	K	E	M	V	I	I	T	R	A	N	T	I	P	L	S	M	I	T	V	V	T	T	S	K	V	H	R	S	I	S

The matrix (M) gene of both KZ and KR AOAV-16 strains was 1242 nt long and encoded a 364 aa M-protein. The M-gene is, in general, the most conserved gene in this genotype, and only one amino acid substitution was found (Table 4). Both AOAV-1 and 16 comprised conserved domain FPIV. Their putative bipartite motif and nuclear localization signal sequences (Aziz-Ul-Rahman et al., 2018) were 247- KKGRKVTFDKLERKLRR -263 in AOAV-1 and 247-KKGKKISFDKLERKIRR-263 in AOAV-16 highly enriched with arginine and lysine residues. Varying positions are underlined. It is worth noting that the M-gene's ORF length was 364 aa and identical for both genotypes.

The large polymerase (L) gene of KZ and KR AOAV-16 strains was 6734 nt long and encoded a 2202 aa L-protein. The L-protein contained 22 amino acids signatures differentiating them (Table 4). In the L gene, four conserved domains I, II, III and IV and ATP binding motif are known in orthoavulaviruses (Aziz-Ul-Rahman et al., 2018). In domains I, II and III, AOAV-1 and 16 strains were almost identical, containing up to two substitutions and they slightly differed by ATP binding motif and domain IV with four aa substitution in each of them (Supplementary Figure S1).

### 3.4 Superficial proteins analysis

The ability of AOAV - 16 Kazakh isolate to cross-react with serum to AOAV-1 indicates not only its genetic but also its antigenic similarity at the level of surface glycoproteins F and HN. Previously, high antigenic relatedness of AOAV-1 and 9 in cross-HI tests to the Korean AOAV-16 was observed (Lee et al., 2017). Comparison of the putative amino acid composition of the surface proteins of these genotypes for the F-gene, in particular for the fusion peptide and even for the hypervariable regions HRa, HRb and HRc (Supplementary Figure S1), showed a very high degree of homology. A high degree of similarity was also found for the HN gene but to a lesser extent than for the F gene.

The fusion (F) gene of KZ and KR AOAV-16 strains was 1823 nt long and encoded an F-protein of 551 aa in length. The putative amino acid sequence of the F-gene cleavage site was defined as LVQAR↓LVG, which typically corresponds to non-pathogenic viruses. AOAV-16 genotype, as well as lentogenic AOAV-1 strains, lack multiple basic residues, and the next amino acid after the cleavage site is leucine. Comparison of the hypervariable regions and the binding peptide of the F protein has also shown high similarity between AOAV- 1 and 16. The F-proteins of both KZ and KR AOAV-16 strains contained seven potential N-linked glycosylation sites with positions that were exactly conserved and located at amino acid 83 of the F2 subunit and 189, 364, 445, 469, 490 and 539 of the F1 subunit (NetNGlyc 1.0).

The HN gene of KZ AOAV-16 isolate was 2041 nt long, but the KR strain had a deletion of 18 nt in the non-coding part. Both viruses encoded a protein of 618 aa length and contained the hexapeptide N-R-K-S-C-S responsible for sialic-acid binding at the cell-surface (Paldurai et al., 2009). The HN-proteins of both AOAV-16 strains contained four potential N-linked glycosylation sites located at amino acids 119, 341, 508 and 602.

Genome alignment of two AOAV-16 strains indicated areas of substantial nt differences distributed unevenly throughout the genome and accounted for 60 putative amino acids.

### 3.5 Phylogenetic analysis of the complete genome and NP-Gene amino acid sequences of published AOAV serotypes

**Figure 1:**
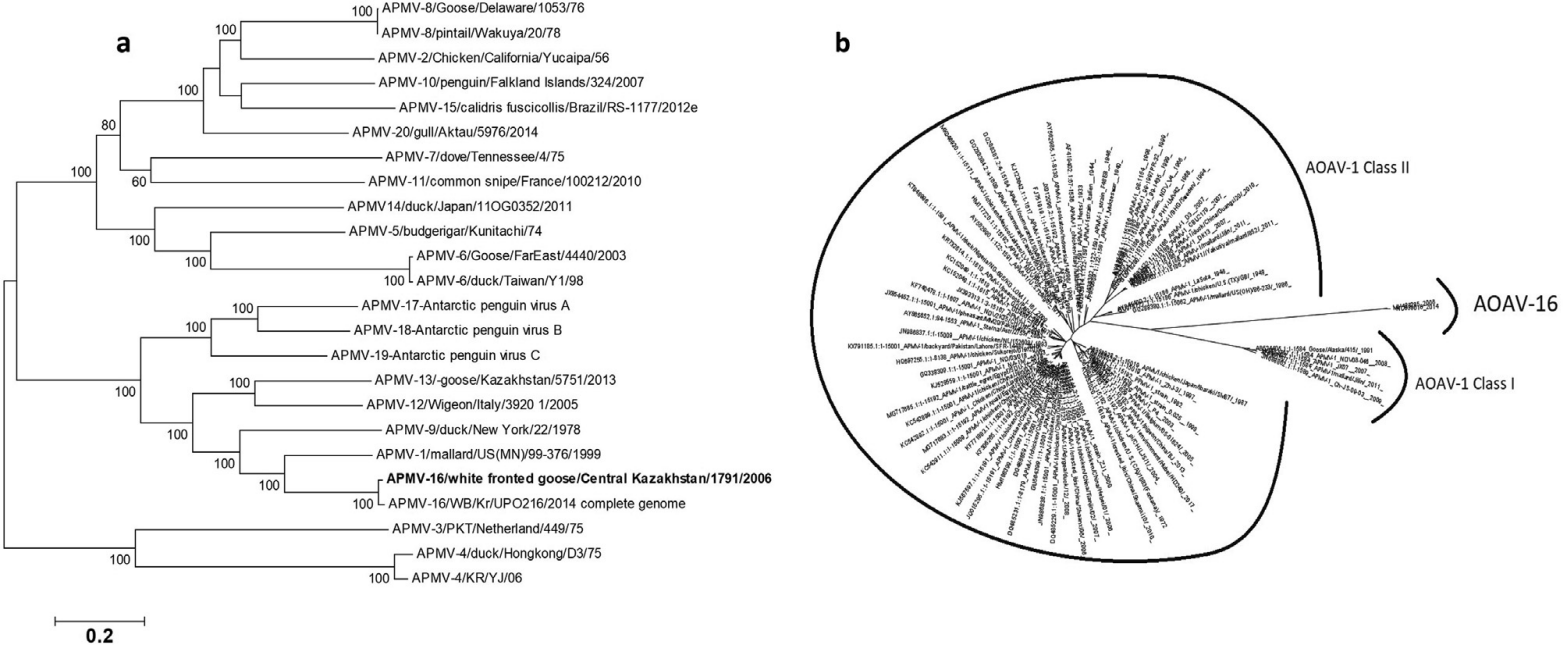
Phylogenetic analyses of the complete genome nucleotide sequences of all the Avian avulavirus species (a) and NP-gene sequences of AOAV-1 and 16 (b).

Phylogenetic trees were generated from the complete genome nucleotide and NP-gene sequence alignments of representative viruses of *Avulavirinae* subfamily (Figure 1). As suggested by International Committee on Taxonomy of Viruses, a new subfamily Avulavirinae was created within the Paramyxoviridae family. Based on the tree topology, three new genera within the subfamily are proposed: Avian orthoavulavirus (AOAV 1, 9, 12, 13, 16, 17, 18 and 19); Metaavulavirus (Avian metaavulaviruses 2, 5, 6, 7, 8, 10, 11, 14, 15 and 20) and Paraavulavirus (Avian paraavulaviruses 3 and 4). The phylogenetic trees here clearly indicate the very close genetic relationship among the KZ and KR AOAV-16 strains, and both of them are closely related to AOAV-1. The AOAV-1 and 16 are clustered within the monophyletic group that contains the representatives of the Orthoavulavirus subfamily.

### 3.6 Selection pressure and molecular clock analyses

The relative numbers of nonsynonymous (dN) to synonymous (dS) substitutions (ratio the dN/dS) per gene (BUSTED) and per site (SLAC,FEL, MEME and FUBAR) were computed to assess selection pressures on AOAV-16 six individual genes. The analysis revealed no evidence for positive selection on gene or site level using all methods, however an abundance of negatively selected sites was found using FEL and FUBAR methods. The P gene appeared to be the most variable gene as it contained the less quantity of negatively selected sites (Supplementary Table S2).

**Figure 2:**
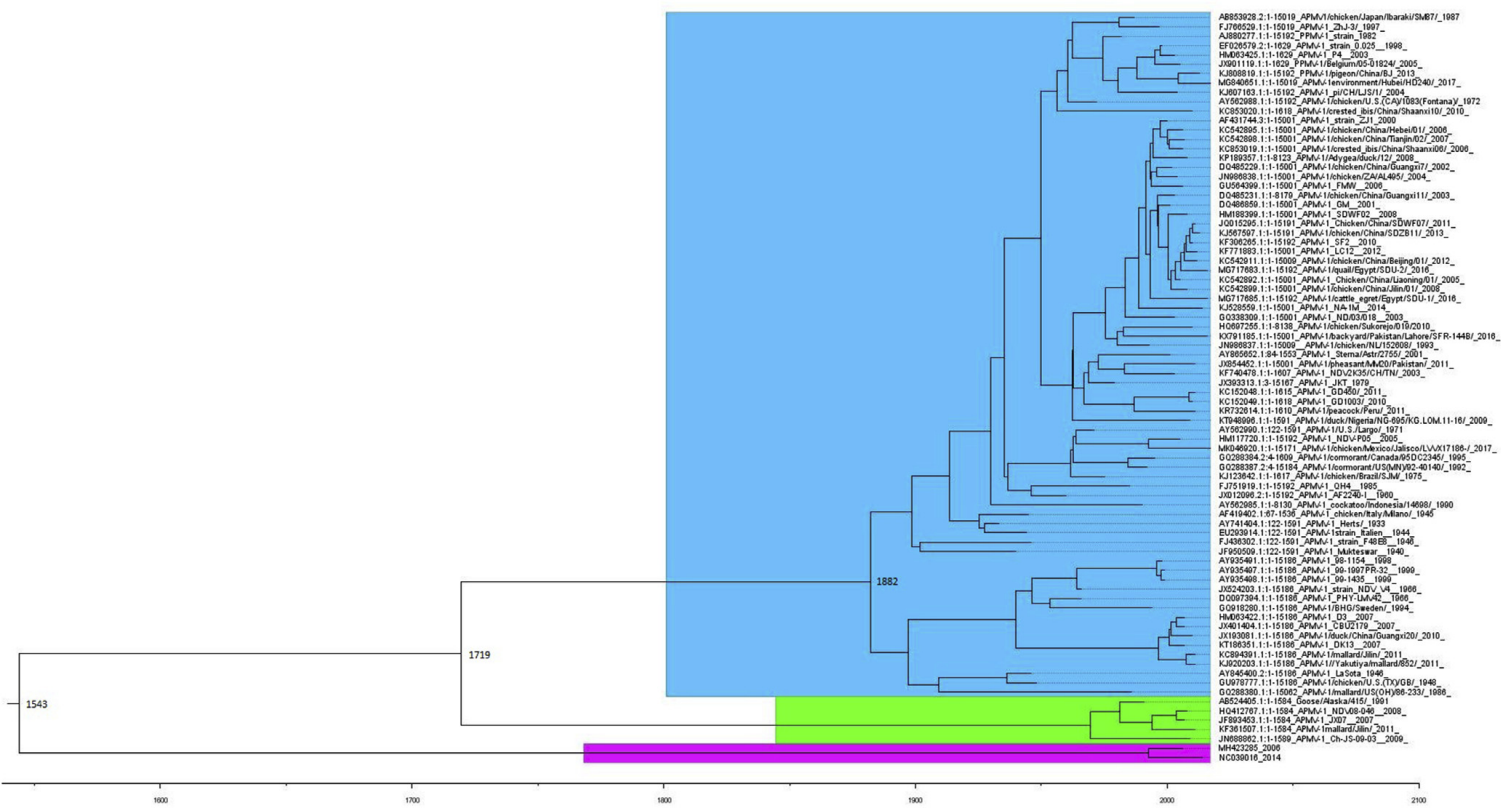
Phylogenetic tree based on the aligned AOAV-1 and 16 NP gene nucleotide sequences. AOAV-1 Class I is coded with green, Class II with light-blue and AOAV-16 with pink.

The nucleoprotein gene was selected for molecular clock estimations because, as an internal gene, it is expected to be one of the most conserved genes in both AOAV 1 (Miller et al., 2009) and AOAV-16 (this research). To be consistent with previous molecular clock research of Newcastle disease virus (Miller et al., 2009) we used the same models: GTR with gamma + invariant along with an uncorrelated relaxed lognormal molecular clock. A coalescent exponential population was set as tree prior. The tMRCA estimates in this research show the divergence between Class II NDV occurred about 1882 that is in agreement with previous reports that established the time of the MRCA for NDV classes II to be around 1883 (Soñora et al., 2015) and 1868–1891 (Chong et al., 2010). Maximum clade credibility trees revealed that class I and class II AOAV-1 have evolved from ancestors that existed around 1719 and this data is close to previously estimated data of 1772–1782 (Taylor et al., 2017). Bayesian estimates indicate that the time of the most recent common ancestor (MRCA) was established to be around 1543 (95% HPD) for AOAV-16 and AOAV-1 species (Figure 2).

## 4 Discussion

Kazakhstan is situated at the center of the Eurasian continent, and its territory is crossed by numerous wild birds’ migration flyways. Hundreds of bird species concentrate in the natural habitats during the migration and breeding periods (Anonimous). Wild avifauna of Central Asia serves as a natural reservoir for many avulavirus species. Seven different species, including avulaviruses 1, 2, 4, 6, 8 and novel AOAV-13 (Yamamoto et al., 2015; Karamendin et al., 2016a; Goraichuk et al., 2016) and Avian metaavulavirus 20 (Karamendin et al., 2017) have been isolated from wild and domestic birds in Central Asia between 1980 and 2014. Extensive distribution of avulaviruses 1, 4 and 6 in Asian subcontinent has been shown (Yamane et al., 1982; Karamendin et al., 2016b).

In 2006, during annual viral surveillance of wild birds in Central Kazakhstan, hemagglutinating agents were isolated from fresh feces collected in the Korgaljyn Nature Reserve. One of those hemagglutinating viruses (currently identified as AOAV-16/white fronted goose/Central Kazakhstan/1791/2006) was classified as an AOAV-1 using the standard HI assay, which has been considered as a standard method for differentiating AOAV species (Alexander, 2000). The results of the current study revealed the failure of using exclusively the classical methods to differentiate different serotypes of AOAVs. Especially for closely related serotypes, cross-reactivities in HI test may cause false grouping or serotyping of AOAV. This phenomenon has been previously shown for the novel avulaviruses 10 and 12, both of which exhibited cross-reactivity with other serotypes (Miller et al., 2010; Terregino et al., 2013). Next generation sequencing allowed us to clarify that the 2006 isolate was a new genotype of AOAVs, and its relationship to the recently discovered AOAV-16 genotype was determined.

The genome of the Kazakh AOAV-16 strain has areas of substantial sequence differences in comparison to Korean strain. These differences were distributed unevenly throughout the genome and accounted for 60 putative amino acids. BLAST search with complete genome sequence has shown an identity of 97 % with Korean strain, and they were most divergent at the P-gene with 94.7% identity, whereas surface proteins (F and HN) were considered as the most variable genes in most other viruses. Most non-synonymous substitutions were found in the P gene and led to the absence of production of W-protein mRNA in the Kazakh strain. The high evolutionary variability of the P gene may be explained via the process of virus adaptation to new hosts (by efficient overcoming of internal protection as interferon production and apoptosis). Two times fewer non-synonymous substitutions in the F and HN genes, which are the envelope surface glycoproteins of the virus and the main targets of the host's immune response, may indicate a minor effect of antigenic drift in the evolution of avulaviruses (Munir et al., 2011).

The study of the evolutionary relationships of AOAV-1 and AOAV-16 showed a very close genetic relationship between these two species, which is also remarkable as high genetic similarity even in hypervariable regions of surface glycoproteins was observed. The previously proposed criterion of 25% divergence in whole genome for distinguishing novel genotype is applicable for AOAV-16, as maximum similarity with AAV-1 is 77–80%. It is obvious that AOAV-1 and AOAV-16 have a common evolutionary origin, and AOAV-16 has the greatest genetic relationship with lentogenic AOAV-1 strains of Class I circulating among wild birds, rather than with velogenic strains of Class II responsible for epizootic outbreaks with high mortality among the domestic birds. MRCA estimates indicate that the divergence time between AOAV-16 and AOAV-1 (MRCA) was established to be around 1543 and further AOAV-1 diverged into two Classes I and II around 1882. Phylogenetic studies also has shown the closer relatedness of Class I to AOAV-16 than Class II.

Genetic analyses of AOAV-16 have shown that the genome of the Korean isolate six years after the isolation of the Kazakh strain, underwent only minor evolutionary changes. This finding is not surprising for avulaviruses, as it was earlier shown that Avian metaavulavirus 8 has revealed little change in its genome after 40 years evolution (Yamamoto et al., 2015). Low evolutionary changes in AOAV-16 potentially indicate its low circulation among wild birds, which is also suggested by only two report of isolation within long period.

We have observed simultaneous circulation of AOAV-1 and AOAV-16 among wild ducks andgeese, and their competition in terms of cross-immunity and cross-neutralization may occur. Although AOAV-1 and 16 were isolated simultaneously in one place from the same flock, genetic recombination between these species was not registered. AOAV-1 has had more success, being a widespread species all over the world and AOAV-16 is a rare species not involved in active circulation, and the possible reason for this can be explained by conducted selection pressure analysis. Previous research (Miller et al., 2009) has revealed positive selection sites in APMV-1 and in this study we revealed absence of them in APMV-16 that means evolutionary advance in APMV-1. Abundance of negative pressure in all genes of APMV-16 was revealed that shows conservative character of the evolutionary process in this species.

## Declarations

### Author contribution statement

Kobey Karamendin: Conceived and designed the experiments; Performed the experiments; Analyzed and interpreted the data; Wrote the paper.

Aidyn Kydyrmanov: Analyzed and interpreted the data.

Yermukhammet Kasymbekov, Aigerim Seidalina, Klara Daulbayeva, Marat Sayatov: Performed the experiments.

Sasan Fereidouni: Analyzed and interpreted the data; Wrote the paper.

### Funding statement

This work was supported by the Ministry of Education and Science of the Republic of Kazakhstan [grant number АР05131549].

### Competing interest statement

The authors declare no conflict of interest.

### Additional information

No additional information is available for this paper.

## Appendix A Supplementary data

Supplementary Table S1 Supplementary Table S1

Supplementary Table S2 The relative numbers of nonsynonymous (dN) to synonymous (dS) substitutions (ratio the dN/dS) per site determined by FEL and FUBAR methods. Supplementary Table S2
