# Supplementary material for: Evolution of Avian orthoavulavirus 16 in wild avifauna of Central Asia
Source: Heliyon. 2020 Jan 7;6(1):e03099. doi: 10.1016/j.heliyon.2019.e03099 (PMC7002782; doi:10.1016/j.heliyon.2019.e03099)
Supplement: Supplementary Figure S1 [file mmc1.docx]

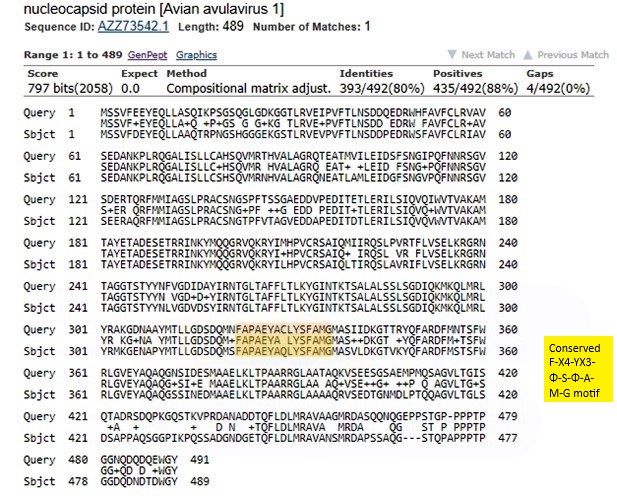

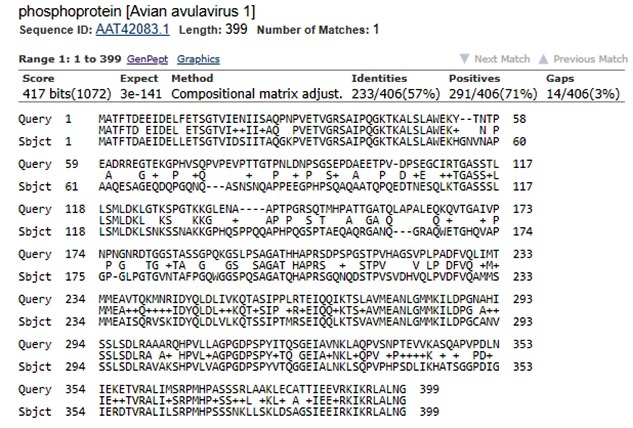

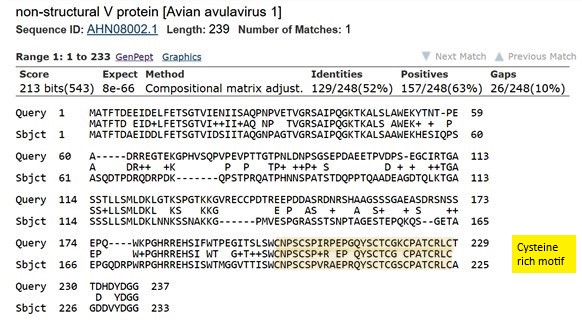


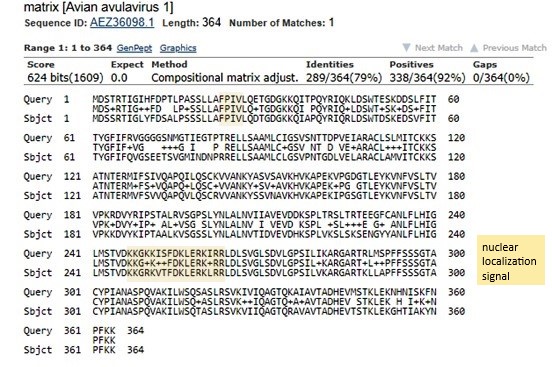


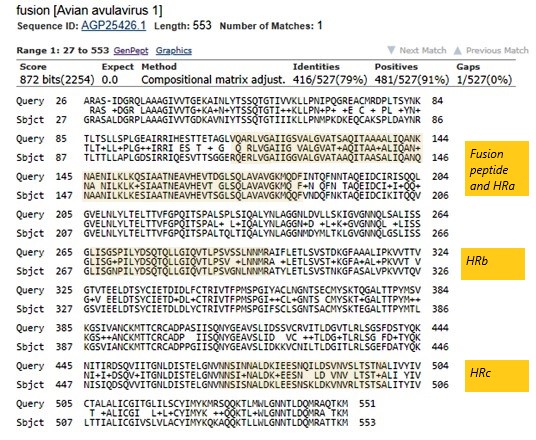


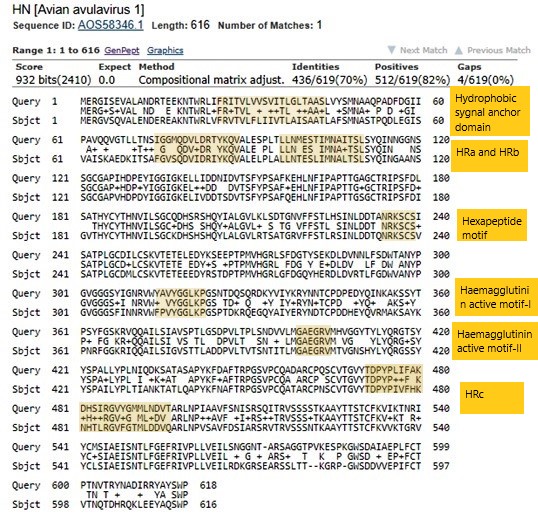


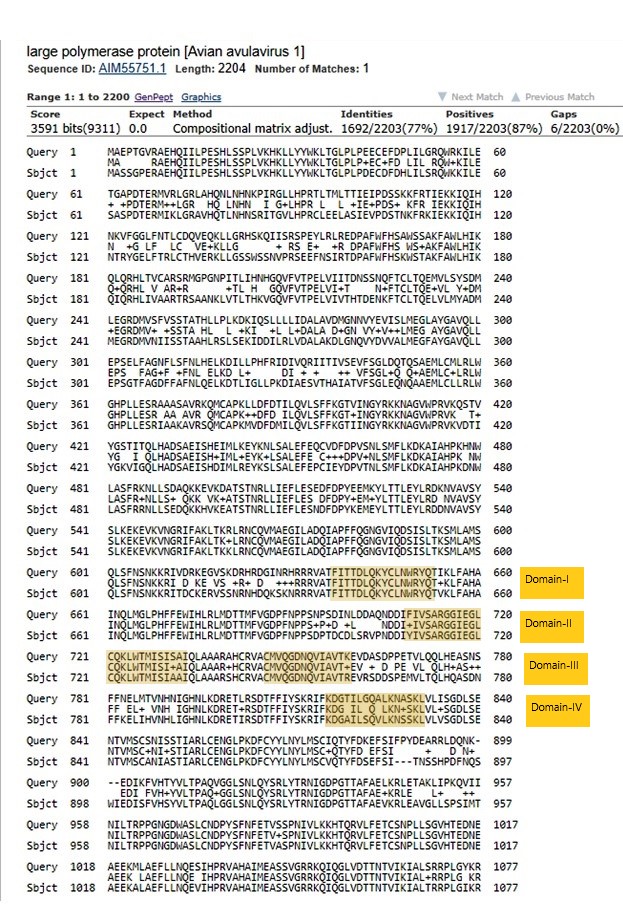


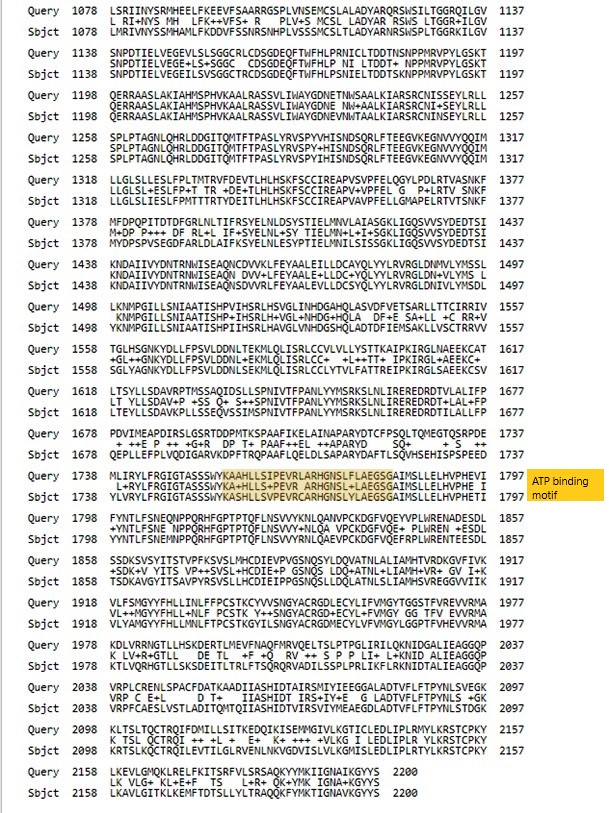


**S1 Fig. Amino acid BLAST search of AOAV-16 sequences that aligned to AOAV-1 proteins**. Motifs are highlighted with yellow
