# Supplementary material for: Evolution of Avian orthoavulavirus 16 in wild avifauna of Central Asia
Source: Heliyon. 2020 Jan 7;6(1):e03099. doi: 10.1016/j.heliyon.2019.e03099 (PMC7002782; doi:10.1016/j.heliyon.2019.e03099)
Supplement: Supplementary Table S1 [file mmc2.docx]

Table S1. Estimates of Evolutionary Divergence at the P protein level between all avian avulavirus species sequences

| Species | AOAV 1 | AMAV  2 | APAV 3 | APAV 4 | AMAV 5 | AMAV 6 | AMAV 7 | AMAV 8 | AOAV 9 | AMAV 10 | AMAV 11 | AOAV 12 | AOAV 13 | AMAV 14 | AMAV 15 | AOAV 16 Kor | AOAV 16 KZ | AOAV Antarctic A | AOAV Antarctic B | AOAV Antarctic C |
| --- | --- | --- | --- | --- | --- | --- | --- | --- | --- | --- | --- | --- | --- | --- | --- | --- | --- | --- | --- | --- |
| AOAV-1/La Sota |  |  |  |  |  |  |  |  |  |  |  |  |  |  |  |  |  |  |  |  |
| AMAV-2/Chicken/California/Yucaipa/56 | 2,497 |  |  |  |  |  |  |  |  |  |  |  |  |  |  |  |  |  |  |  |
| APAV-3/PKT/Netherland/449/75 | 2,472 | 2,414 |  |  |  |  |  |  |  |  |  |  |  |  |  |  |  |  |  |  |
| APAV-4/duck/Hongkong/D3/75 | 2,517 | 2,723 | 2,455 |  |  |  |  |  |  |  |  |  |  |  |  |  |  |  |  |  |
| AMAV-5/budgerigar/Kunitachi/74 | 2,339 | 2,108 | 2,964 | **2,987** |  |  |  |  |  |  |  |  |  |  |  |  |  |  |  |  |
| AMAV-6/duck/HongKong/18/199/77 | 2,440 | 2,259 | 2,808 | 2,663 | 1,709 |  |  |  |  |  |  |  |  |  |  |  |  |  |  |  |
| AMAV-7/dove/Tennessee/4/75 | 2,533 | 2,026 | 2,617 | 2,816 | 2,204 | 1,947 |  |  |  |  |  |  |  |  |  |  |  |  |  |  |
| AMAV-8/Goose/Delaware/1053/76 | 2,488 | 1,225 | 2,739 | 2,748 | 1,978 | 1,920 | 2,188 |  |  |  |  |  |  |  |  |  |  |  |  |  |
| AOAV-9/duck/New_York/22/1978 | 0,933 | 2,407 | 2,324 | 2,353 | 2,486 | 2,469 | 2,526 | 2,461 |  |  |  |  |  |  |  |  |  |  |  |  |
| AMAV-10/penguin/Falkland_Islands/324/2007 | 2,428 | 1,173 | 2,544 | 2,857 | 2,258 | 1,759 | 1,834 | 1,057 | 2,557 |  |  |  |  |  |  |  |  |  |  |  |
| AMAV-11/common_snipe/France/100212/2010 | 2,296 | 2,017 | 3,516 | 2,600 | 2,096 | 1,993 | 2,183 | 1,950 | 2,009 | 1,736 |  |  |  |  |  |  |  |  |  |  |
| AOAV-12/Wigeon/Italy/3920_1/2005 | 0,935 | 2,531 | 2,806 | 2,675 | 2,294 | 2,483 | 2,643 | 2,396 | 1,028 | 2,765 | 1,931 |  |  |  |  |  |  |  |  |  |
| AOAV-13/goose/Kazakhstan/5751/2013 | 0,991 | 2,490 | 2,454 | 2,787 | 2,328 | 2,448 | 2,641 | 2,476 | 1,007 | 2,755 | 2,216 | 0,643 |  |  |  |  |  |  |  |  |
| AMAV -14/duck/Japan/11OG0352/2011 | 2,424 | 2,195 | 2,749 | 2,615 | 1,675 | 1,380 | 2,345 | 1,876 | 2,236 | 1,852 | 2,096 | 2,228 | 2,168 |  |  |  |  |  |  |  |
| AMAV -15/calidris_fuscicollis/Brazil/RS-1177/12 | 2,241 | 1,375 | 2,848 | 2,655 | 2,060 | 2,014 | 2,523 | 1,267 | 2,337 | 1,244 | 1,751 | 2,326 | 2,538 | 2,036 |  |  |  |  |  |  |
| AOAV-16//WB/Korea/UPO216/2014 | **0,553** | 2,275 | 2,446 | 2,411 | 2,397 | 2,423 | 2,475 | 2,345 | 0,976 | 2,326 | 2,095 | 0,958 | 0,974 | 2,372 | 2,363 |  |  |  |  |  |
| AOAV-16/w-f goose/Central Kazakhstan/1791/2006 | **0,558** | 2,366 | 2,460 | 2,373 | 2,450 | 2,515 | 2,499 | 2,375 | 0,997 | 2,347 | 2,106 | 0,945 | 0,979 | 2,345 | 2,388 | 0,033 |  |  |  |  |
| AOAV-17/Antarctic_penguin_virus_A | 1,376 | 2,484 | 2,531 | 2,776 | 2,446 | 2,478 | 2,385 | 2,370 | 1,497 | 2,705 | 2,648 | 1,399 | 1,325 | 2,271 | 2,461 | 1,263 | 1,232 |  |  |  |
| AOAV-18/Antarctic_penguin_virus_B | 1,290 | 2,637 | 2,497 | 2,702 | 2,467 | 2,342 | 2,535 | 2,613 | 1,506 | 2,766 | 2,425 | 1,201 | 1,275 | 2,316 | 2,617 | 1,270 | 1,277 | 0,684 |  |  |
| AOAV-19/Antarctic_penguin_virus_C | 1,314 | 2,483 | 2,431 | 2,804 | 2,282 | 2,899 | 2,308 | 2,457 | 1,598 | 2,507 | 1,955 | 1,399 | 1,326 | 2,403 | 2,457 | 1,412 | 1,410 | 0,933 | 0,890 |  |
| AMAV -20/gull/Kazakhstan/5976/2014 | 2,199 | 1,215 | 2,465 | 2,541 | 1,805 | 1,871 | 2,002 | 1,124 | 2,153 | 1,198 | 1,720 | 2,042 | 2,194 | 1,571 | 1,286 | 2,332 | 2,333 | 2,362 | 2,470 | 2,301 |

AOAV - Avian orthoavulavirus, AMAV - Avian metaavulavirus, APAV - Avian paraavulavirus.

The number of amino acid substitutions per site between sequences are shown. Bald values in shadowed cells are maximum and minimal interspecies distances.
